# Cataract surgery in neovascular AMD: impact on visual acuity and disease activity

**DOI:** 10.1186/s12886-023-03028-7

**Published:** 2023-06-16

**Authors:** Hin Yan Tang, Mats Rosén, Elisabet Granstam

**Affiliations:** 1 Department of Ophthalmology, County Hospital of Västmanland, Västerås, Sweden; 2 Centre for Clinical Research, Region Västmanland – Uppsala University, S-721 89 Västerås, Sweden

**Keywords:** Neovascular AMD, Anti-VEGF treatment, Cataract surgery, Distance visual acuity, Near visual acuity, Optical coherence tomography (OCT)

## Abstract

**Background:**

Cataract and neovascular age-related macular degeneration (nAMD) often co-exist and both contribute to impaired vision. It has been debated whether cataract surgery can increase nAMD activity. The purpose of this retrospective study was to investigate the impact of cataract surgery on visual acuity, treatment intensity for nAMD and macular morphology in patients with on-going treatment for nAMD.

**Methods:**

Data was obtained from the Swedish Macular Register, the Swedish National Cataract Register, optical coherence tomography (OCT) images and patient charts. All eyes were treated at the Department of Ophthalmology at the County Hospital of Västmanland, Västerås, Sweden. Follow-up was 6 months after surgery. The study was approved by the Swedish Ethical Review Authority.

**Results:**

In total, 156 patients (168 eyes) were included. The mean age at cataract surgery was 82 (standard deviation, SD 6) years. Both distance and near visual acuity improved after surgery. Distance visual acuity increased from 59 (SD 12) to 66 (SD 15) letters ETDRS (*P* < 0.001). Proportion of eyes with normal near visual acuity increased from 12 to 41%. The anti-vascular endothelial growth factor (VEGF) treatment intensity remained unchanged: mean of 3.4 (SD 1.9) and 3.3 (SD 1.7) treatments were given 6 months pre- and postoperatively, respectively. The prevalence of intraretinal fluid (IRF) in the macula increased from 22 to 31% postoperatively, while subretinal fluid, fluid under the pigment epithelium (sub-RPE fluid) and central retinal thickness were unaltered. In eyes with new IRF, improvement in visual acuity and number of anti-VEGF treatments were similar to eyes without new IRF.

**Conclusion:**

Cataract surgery improved visual acuity in patients with on-going treatment for nAMD and did not affect anti-VEGF treatment intensity. Macular morphology remained unchanged. The slight increase in intraretinal fluid after surgery was not found to affect visual acuity or anti-VEGF treatment intensity. It is hypothesized that this might indicate that it represents degenerative intraretinal cystic fluid.

## Key messages

• It has been demonstrated that cataract surgery in eyes with nAMD improves distance visual acuity

• In this study in eyes with on-going anti-VEGF treatment for nAMD, cataract surgery improved both distance and near visual function.

• Cataract surgery did not affect anti-VEGF treatment intensity over 6 months postoperatively.

• A slight increase in new intraretinal fluid, which was observed at 6 months, did not affect neither the visual outcome nor the anti-VEGF treatment intensity after surgery and possibly represents degenerative intraretinal cystic fluid.

## Background

Age-related cataract is the leading cause of blindness among the global population [1] and a leading contributor of visual impairment. It frequently co-exists with age-related macular degeneration (AMD) [2]. AMD is the most common cause of severe visual impairment in people over 60 years of age in European countries, and the prevalence seems to increase [3].

Cataract is treated surgically by removing the opaque lens [4]. Surgery generally results in improved visual acuity and it is one of the most common surgical procedures performed. Improvements of the surgical techniques and intraocular lens replacement technology have further contributed to the success of cataract surgery [4]. Neovascular AMD (nAMD) is treated with intravitreal anti-vascular endothelial growth factor (anti-VEGF) injections. Treatment is repeated over long periods of time, often life-long, and has significantly improved the outcome of nAMD [5].

Presence of cataract in patients with nAMD contributes to the visual impairment of the patient. Performing cataract surgery to obtain best possible visual function for the patient is often advocated. Further, cataract may reduce visibility of the retina and attenuates the quality of optical coherence tomography (OCT) imaging, leading to less reliable assessment of the macula. This might also strengthen the indication to undergo cataract extraction.

Cataract surgery in patients with ongoing anti-VEGF treatment for nAMD has been shown to induce significant improvements in distance visual acuity [6]. However, concerns have been raised that cataract surgery might increase the risk of progression of preexisting AMD [7, 8] potentially by inducing an inflammatory response. Increased incidence of new or worse cystoid changes on OCT after cataract surgery was observed in one study in 40 eyes with on-going treatment for nAMD [9], suggesting increased nAMD activity after surgery. In contrast, pre-operative macular fluid on OCT remained unchanged after cataract extraction in 23 nAMD eyes receiving anti-VEGF treatment [10], indicating stable nAMD disease activity after surgery. In a register study of 111 eyes with nAMD, central macular thickness was even found to decrease following cataract operation [11]. At present, there is still lack of strong evidence-based guidelines regarding the safety and timing of cataract extraction in patients with nAMD on active anti-VEGF treatment [7].

The aim of this study was to investigate the effect of cataract surgery on the visual acuity, macular anatomy on OCT and injection treatment intensity in a larger group of patients with ongoing anti-VEGF treatment for nAMD.

## Methods

This study is a retrospective registry-based observational study of cataract surgery performed in patients with nAMD at the Department of Ophthalmology at the county hospital of Västmanland in Västerås, Sweden. Data was obtained from the Swedish Macular Register (SMR), National Cataract Register (NCR), patient charts and from the optical coherence tomography (OCT)-imaging (Topcon Corporation, Tokyo, Japan). The study was approved by the Swedish Ethical Review Authority (Dnr 2020–00249) and adhered to the tenets of the Declaration of Helsinki.

The primary outcome was number of anti-VEGF treatments during 6 months before and 6 months after surgery, respectively. Secondary outcomes were changes in visual acuity at distance and near and change in macular status regarding intraretinal fluid (IRF), subretinal fluid (SRF), sub-retinal pigment epithelial fluid (sub-RPEF) and central retinal thickness (CRT) from at the time of surgery to 6 months after cataract surgery.

Patients were identified by combining the data from the SMR with data from the NCR regarding treated patients in Västmanland county. Patients with at least one registration in both registers during the same year were identified. Registrations from 2010 until August 31, 2020 were collected.

For inclusion in this study the registrations in SMR and NCR had to involve the same eye, have the diagnosis nAMD registered in SMR at least 6 months before cataract surgery and have treatment or follow-up registrations in SMR during a period of at least 6 months before and 6 months after the cataract surgery. Anti-VEGF treatment for nAMD could be either according to treat and extend (TE) regimen or as needed, pro re nata (PRN) as described in detail previously [12]. Indication for cataract surgery was presence of significant symptomatic cataract and/or cataract precluding adequate OCT imaging of the macula. Preferred macular status was dry on stable treatment interval. Patients registered in SMR because of other diagnosis such as diabetic macular edema or macular edema secondary to retinal vein occlusion were excluded from the study as well as patients with onset of nAMD less than 6 months before cataract surgery.

Data regarding date of cataract surgery was collected from the NCR. From SMR the following data was collected: distance and near visual acuity in the affected eye at 6 months before and 6 months after cataract surgery, the number of anti-VEGF injections in the period 6 months pre- and 6 months post- cataract surgery, respectively as well as total number of anti-VEGF injections. The data regarding visual acuity and treatment intensity collected from the SMR were compared with patient charts for validation The conformity of data between SMR and patient charts regarding anti-VEGF injections was 82% preoperatively and 87% postoperatively which was considered unacceptable. Therefore, the number of intravitreal anti-VEGF injections was collected from patient charts. In cases where the follow-up interval was extended, the visual acuity nearest 6 months preoperatively and postoperatively was chosen. Information about gender, treatment regimen, age at cataract surgery, complications during surgery and post-operative inflammation was collected from patient records.

Best corrected visual acuity (BCVA) was measured using the Early Treatment Diabetic Retinopathy Study (ETDRS) letter chart at 2 m. Near visual acuity was examined using the Jaeger eye chart in Swedish. The text sizes on the chart are converted into points and have the following values: 5, 6, 8, 10, 12, 14, 18 and 24p where 5p is the best near visual acuity [13].

OCT images taken 6 months preoperatively and 6 months postoperatively (OCT 2000, Topcon Corporation, Tokyo, Japan) were collected from the OCT-database IMAGEnet and analyzed. The presence or absence of intraretinal fluid (IRF), subretinal fluid (SRF), and sub-retinal pigment epithelial fluid (sub-RPEF) was assessed and central retinal thickness (CRT) was measured using the macular thickness map protocol. The variables related to fluid were defined according to Schmidt-Erfurth et al. 2015 [14]. IRF was defined as round, minimally reflective spaces (cysts) within the neurosensory retina. SRF was defined as a nonreflective space between the posterior boundary of the neurosensory retina and the retinal pigment epithelium (RPE)/choriocapillaris signal. Sub-RPE fluid was defined as a focal elevation of the reflective RPE band over an optically clear or moderately reflective space with a minimum width of 400 um at the base or a minimum height of 200 um from the surface of the RPE band to the surface of the choriocapillaris [14].

### Sample size calculation

For sample size calculation, the results from Kessel et al. 2016 were used [15]. The mean number of anti-VEGF injections given was 1.5 (range 1–5) in the 6 months before surgery and 1.7 (range 1–4) in the 6 months after surgery. We found that a sample size of 186 eyes was required to detect a significant difference between measurements with a significance level of *P* < 0.05 and a power of 80%.

### Statistical analysis

Statistical analyses were performed using SPSS. Student’s t test for paired and unpaired data was used as appropriate. *P* value < 0.05 was considered statistically significant. Data are presented as mean and standard deviation (SD) unless otherwise specified. Snellen acuity is presented as median.

## Results

**Fig. 1:**
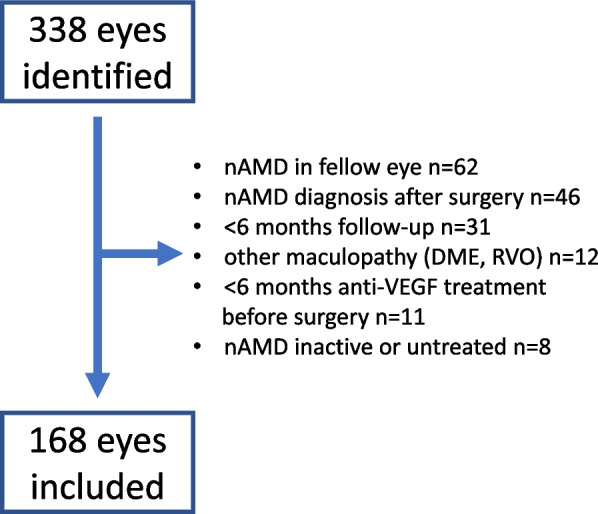
Flow-chart for inclusion into the study. Eyes were identified by combining the data from the Swedish Macular Register (SMR) with data from the National Cataract Register (NCR) regarding treated patients in Västmanland county, Sweden

In total 338 eyes (297 individuals) were identified when matching the SMR with the NCR. After applying exclusion criteria, 168 eyes (156 individuals) were included in the study (Fig. 1). Background demographic data for the study cohort are presented in Table 1.

**Table 1:** Demographic data of study cohort

Variable	Value	Range
No of patients	156	
Gender (proportion female, %)	65 (102/156)	
No of eyes	168	
Age at surgery (years; mean, SD)	82 (6)	
Treatment for nAMD before surgery (years; median, range)	6.1	0.5–10.6
Total no of IVT before surgery (median; range)	32	3–75

### Visual acuity

**Fig. 2:**
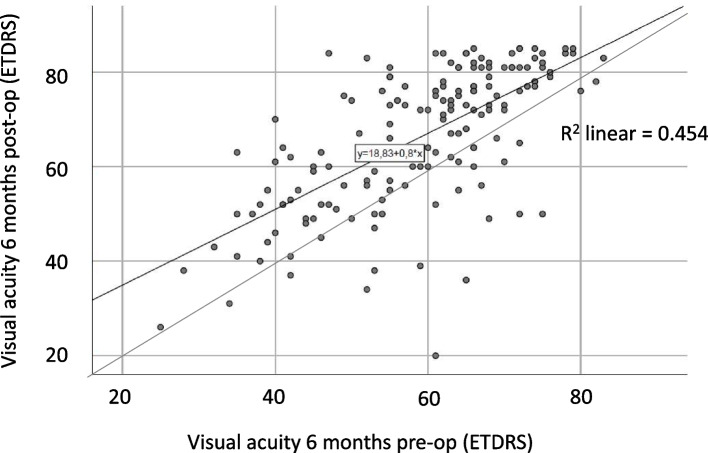
Association between visual acuity 6 months before cataract surgery (pre-op; x-axis) and visual acuity 6 months after surgery (post-op; y-axis). A higher ETDRS score preoperatively was associated with a better ETDRS score postoperatively with a correlation coefficient R2 = 0.454

The best corrected visual acuity (ETDRS letter score) increased from 59 (SD 12) letters ETDRS 6 months preoperatively to 66 (SD 15) letters ETDRS 6 months postoperatively (*P* < 0.001). The distribution of visual acuity ETDRS letter score pre- and postoperatively is shown in Fig. 2. A higher ETDRS score preoperatively was associated with a better ETDRS score postoperatively with a correlation coefficient R2 = 0.454. Multiple linear regression analysis revealed a significant association between pre- and postoperative ETDRS score (*P* < 0.001) whereas duration of nAMD-treatment preoperatively was not related to postoperative ETDRS score (*P* = 0.380).

**Fig. 3:**
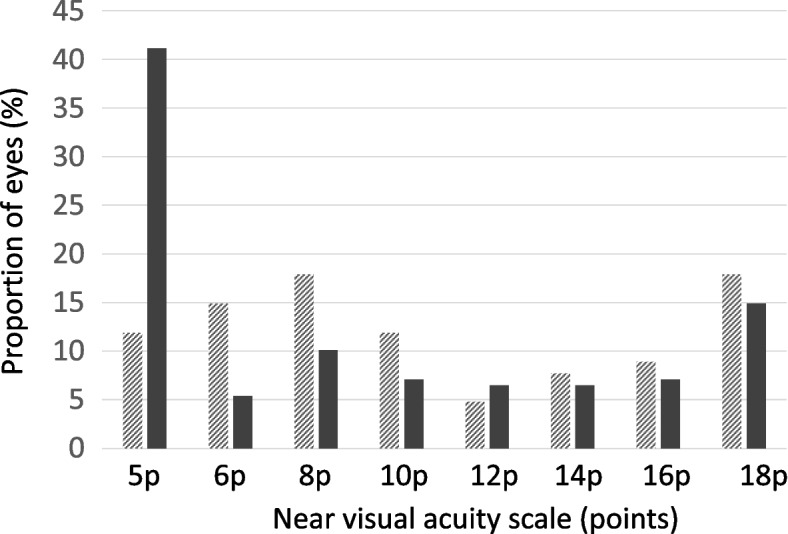
Proportion of eyes (%) with different levels of near visual acuity 6 months before cataract surgery (hatched bars) and 6 months after surgery (filled bars). Near visual acuity was measured on a non-continuous scale ranging from 5 to 18 p

The median near visual acuity improved from 10 p (interquartile IQ range 6–18 p) 6 months before surgery to median 8 p (IQ range 5–12 p). Overall, 85% had either the same or improved near visual acuity 6 months post-surgery. The distribution of near visual acuity along the visual acuity scale 6 months before and after cataract surgery are shown in Fig. 3. The proportion of eyes with normal near visual acuity of 5p increased postoperatively from 12 to 41%.

### Anti-VEGF treatment

A mean of 3.4 (SD 1.9) anti-VEGF injections were administered during 6 months before cataract surgery and 3.3 (SD 1.7) injections during 6 months after surgery (*P* = 0,269).

### OCT

**Fig. 4:**
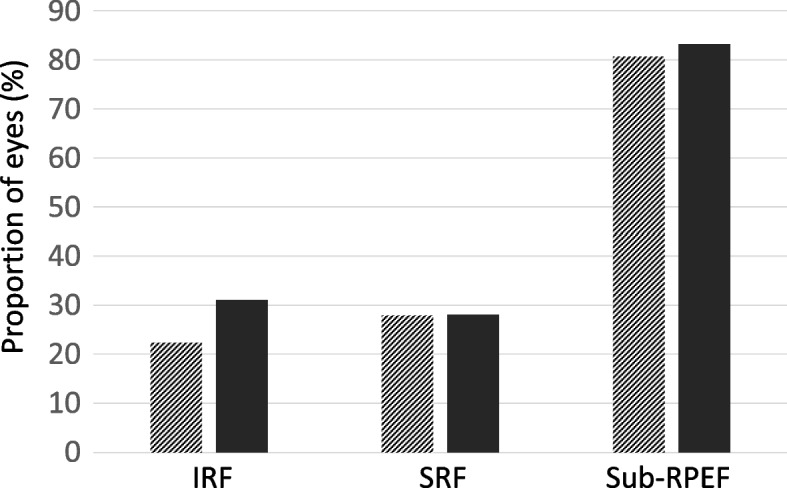
Proportion of eyes (%) with intraretinal fluid (IRF), subretinal fluid (SRF) and sub-retinal pigment epithelial fluid (sub-RPEF) on OCT 6 months before cataract surgery (hatched bars) and 6 months after surgery (filled bars)

Qualitative evaluation of OCT images obtained 6 months pre- and 6 months postoperatively showed that CRT was unchanged with mean 223 (SD 61) before surgery and 223 (SD 48) after surgery (*P* = 0.915). The proportion of eyes with IRF, SRF and sub-RPEF are presented in Fig. 4. The proportion of eyes with IRF increased from 22% pre- to 31% 6 months postoperatively (*P* = 0.023) while SRF and sub-RPEF remained unchanged (*P* = 0.873 and *P* = 0.249 respectively).

In 23 eyes, new IRF developed on OCT after cataract surgery. Cataract extraction improved visual acuity in these eyes from 58 (SD 14) to 67 (SD 15) letters ETDRS (*P* < 0.001), which was similar to the change in visual acuity in eyes without new IRF after surgery: from 59 (SD 12) to 66 (SD 15; *P* < 0.001) letters ETDRS. The number of anti-VEGF injections given remained stable during 6 months before and after cataract surgery, 2.6 (SD 1.6) and 3.0 (SD 1.5), respectively in eyes with new IRF (*P* = 0.281). Overall, 11% of eyes had signs of inflammation in the anterior segment postoperatively. Sub-group analysis showed that the proportion of eyes with intraocular inflammation was similar in eyes with new IRF (13%) as compared with eyes without new IRF (11%).

Among eyes with new IRF, 65% were treated according to TE whereas among eyes without new IRF, 76% were treated according to TE. This difference was not statistically significant (*P* = 0.070) although in favor of TE as treatment regimen. Further, the group with newly developed IRF was compared to the group without new IRF with regards to age, previous period of treatment, previous number of injections and ETDRS score before surgery. No statistically significant differences were found between groups.

### Complications

Cataract surgery was complicated in two eyes. There was posterior capsule rupture in one eye, which had been treated with 11 intravitreal anti-VEGF-injections prior to cataract surgery. One eye had secondary surgery due to remaining cortex. All surgical procedures were finished uneventfully. There were no reports of endophthalmitis.

## Discussion

In this study we found that cataract surgery in patients with on-going anti-VEGF treatment for nAMD significantly increased both distance and near visual acuity. Treatment intensity for underlying nAMD was not affected. The macular status remained unchanged besides a slight increase in IRF.

The finding of improvement in distance visual acuity following cataract surgery in nAMD patients is in line with previous studies. Secondary analyses from the ANCHOR/MARINA studies using ranibizumab for treatment of nAMD demonstrated increase in visual acuity after cataract surgery [6]. More recent register-based studies [11, 16] as well as smaller, retrospective cohort studies have reported similar findings [9, 15]. In our study we also found significant improvements in near visual acuity. More than 40% of the patients reported normal near visual acuity of 5 p, which would allow reading regular newspaper text. Good near visual acuity has been found to have an important impact on health-related quality of life especially in patients with nAMD [17] and is considered an important finding in the present study.

In our study, we found that a higher ETDRS letter score preoperatively was associated with a better ETDRS letter score postoperatively. Other studies have reported larger improvements in visual acuity in patients with worse vision before cataract surgery [16, 18]. It seems likely that the potential for improvement in visual acuity is larger in patients with low visual acuity preoperatively but that the final outcome of surgery is dependent on visual function before surgery. This applies also to visual outcome of anti-VEGF treatment for nAMD [19].

In the present study, the mean number of anti-VEGF injections remained stable 6 months after cataract surgery compared with 6 months before surgery, which is in line with previous smaller studies [9, 15, 20]. In the register-based study by Karesvuo et al. 2021 [11], slightly longer anti-VEGF treatment intervals were observed after cataract surgery. Similar indications of reduced need for anti-VEGF treatment over time was observed by Tabandeh et al. 2012 [21] and Choi et al. 2021 [22]. On the other hand, in a matched case–control study the proportion of eyes with active nAMD eyes which did not have cataract extraction declined over time whereas in nAMD eyes subjected to cataract surgery, the proportion of eyes with active nAMD remained unchanged indicating some increase in disease activity after surgery [16]. In our study, treatment for nAMD had been on-going for more than 6 years at the time of cataract surgery, which was longer compared to other studies [11, 15, 16, 20–22] further emphasizing our conclusion that cataract surgery does not seem to affect chronic, stable treated nAMD.

We found a slight increase in prevalence of intraretinal fluid 6 months after cataract surgery. In our study, improvement in visual acuity after cataract extraction was comparable between eyes with and without new IRF and the frequency of anti-VEGF treatments was not affected, indicating that this IRF represented degenerative cystic intraretinal fluid. In line with our findings are results from the study by Saraf et al. 2015 [9] where increase in cystoid retinal changes on OCT after cataract surgery was observed without affecting neither intensity of anti-VEGF treatment nor visual outcome. Performance of fluorescein angiography could have been of value to determine the status of nAMD disease at this point. Longer follow-up might be of importance to further elucidate the clinical significance of this finding. Another possible explanation is that the increase in IRF might represent post-operative macular edema. The incidence of postoperative macular edema in uncomplicated cataract surgery in general is 1.17% [23] and pre-operative age-related macular degeneration has not been found to increase the risk of post-operative macular edema [23]. However, given that the peak incidence of postoperative macular edema occurs at 6–8 weeks after surgery [24] it seems less likely that our finding at 6 months could be related to the surgical procedure.

The timing of cataract surgery in eyes with on-going anti-VEGF treatment for nAMD has been discussed. Patients undergoing cataract surgery within 6 months from start of anti-VEGF treatment were found more likely to lose rather than gain visual acuity after surgery [16], suggesting that cataract surgery should be avoided during the first six months after initiating therapy for nAMD. On the other hand, previous intravitreal injections have been found to increase the risk for intraoperative complications during cataract surgery [25]. Since the risk was found to be related to number of injections [25], possibly due to repeated iatrogenic micro-damage to the lens or zonulae, the finding suggests that cataract extraction must not be postponed too long. Although our patients had been on active anti-VEGF treatment for median 6 years and had received up to 75 injections at the time of surgery posterior capsule rupture occurred in only one eye.

The strength of our study is that it was based on a large number of patients and included all eyes with wet AMD registered in SMR that had undergone cataract surgery from a single clinical setting. Although we did not fully reach the calculated sample size, we consider our conclusion that cataract surgery did not induce any change in need for anti-VEGF treatment for nAMD robust and that addition of another 18 eyes would not change this. One limitation of this study is absence of a matched control group that received anti-VEGF therapy not undergoing cataract surgery. Further, no cataract grading classification was applied. Another limitation is the retrospective nature of this study and limited follow-up. Longer follow-up would give further insights especially in the nature and potential impact of increase in IRF identified.

## Conclusion

Cataract surgery was found to improve both distance and near visual function in the majority of patients with on-going anti-VEGF treatment for nAMD without worsening of underlying nAMD.

## Data Availability

All data is available through the SMR and upon request from Region Västmanland, Västerås, Sweden.

## Abbreviations

ANCHOR: The Anti-VEGF Antibody for the Treatment of Predominantly Classic Choroidal Neovascularization in Age-Related Macular Degeneration Study
AMD: Age-Related Macular Degeneration
BCVA: Best Corrected Visual Acuity
CRT: Central Retinal Thickness
DME: Diabetic Macular Edema
Dnr: Diary Official Registration Number
ETDRS: Early Treatment Diabetic Retinopathy Study
IRF: Intraretinal Fluid
IVT: Intravitreal Treatment
IQ: Interquartile
MARINA: The Minimally Classic/Occult Trial of the anti-VEGF Antibody Ranibizumab in the Treatment of Neovascular Age-Related Macular Degeneration Study
nAMD: Neovascular Age-Related Macular Degeneration
No: Number
NCR: Swedish National Cataract Register
OCT: Optical Coherence Tomography
p: Typographical Point
P: Probability Value
PRN: Pro Re Nata
RPE: Retinal Pigment Epithelium
RVO: Retinal Vein Occlusion
SD: Standard Deviation
SMR: Swedish Macular Register
SRF: Subretinal Fluid
SPSS: Statistical Package for the Social Sciences
Sub-RPEF: Sub-Retinal Pigment Epithelial Fluid
TE: Treat and Extend
